# Pharmacogenetic biomarkers for predicting therapeutic response to JAK inhibitors in rheumatoid arthritis

**DOI:** 10.1515/almed-2025-0180

**Published:** 2026-04-23

**Authors:** Chamaida Plasencia-Rodríguez, Marta Novella-Navarro, Juan Luis Valdivieso Shephard, Ana Martínez-Feito, Keren Reche-Yebra, Manuel Juárez-García, Alejandro Villalba-Yllán, Tania S. Rubio-Lepe, Pedro Arias, Victoria Lucia Collada Sánchez, Susana Martin-López, Itsaso Losantos-García, Alberto M. Borobia, Rocío Rosas-Alonso

**Affiliations:** Rheumatology Department, La Paz University Hospital, Madrid, Spain; Pharmacogenetics Unit, La Paz University Hospital, Madrid, Spain; Immunology, La Paz University Hospital, Madrid, Spain; Servicio de Farmacia Hospitalaria, La Paz University Hospital, Madrid, Spain; Biostatistics Platform, La Paz University Hospital Research Institute (IdiPAZ), Madrid, Spain; Biomarkers and Experimental Therapeutics in Cancer, La Paz University Hospital Research Institute (IdiPAZ), Madrid, Spain

**Keywords:** rheumatoid arthritis, JAK inhibitors, pharmacogenetics

## Abstract

**Objectives:**

Rheumatoid arthritis (RA) is a chronic inflammatory disease of immune-mediated origin. Janus kinase inhibitors (JAK inhibitors) have expanded therapeutic options in recent years; however, clinical response varies among patients, and no biomarkers are currently available to predict their efficacy. The aim of this study was to analyze the association between pharmacogenetic variants and clinical response to JAK inhibitors in patients with RA.

**Methods:**

An observational, ambispective, single-center study was conducted in adult patients diagnosed with RA and treated with JAK inhibitors. Sociodemographic and clinical characteristics were collected from electronic medical records. Five genetic variants previously associated with response to biological therapies in RA were analyzed through genotyping.

**Results:**

A total of 56 patients were included in the study. A statistically significant association was observed between the rs20575 variant of the *TNFRSF10A* gene and clinical remission, with CG or CC genotypes being more frequent among patients who achieved remission at the last available follow-up. Additionally, the rs1801274 polymorphism of the *FCGR2A* gene was associated with a lower likelihood of achieving low disease activity or remission in patients with the AA genotype, both at 12 months and at the last available follow-up.

**Conclusions:**

This study highlights the potential of pharmacogenetics to personalize RA treatment by establishing, for the first time, that specific genetic variants involved in immune response may significantly influence the efficacy of JAK inhibitors. These findings open new perspectives for optimizing therapeutic decisions, although further studies are needed to validate them.

## Introduction

Rheumatoid arthritis (RA) is a chronic immune-mediated inflammatory disease characterized by joint-related clinical manifestations with structural damage, but it also presents systemic extra-articular features [1], 2].

The global prevalence of RA is estimated to be between 0.5 and 1 %, with an age-related increase and a higher incidence in women. [3]. Various immune system cells and multiple effector pathways are involved in the cascade of events that promote the development and progression of the disease [4], 5].

In recent years, advances in understanding the immunopathogenic mechanisms underlying RA have driven the development of new therapeutic strategies. Among these, biological disease-modifying antirheumatic drugs (bDMARDs) and targeted synthetic DMARDs (tsDMARDs) stand out. Currently, the most commonly used bDMARDs as first-line treatment are tumor necrosis factor inhibitors (TNFi), while tsDMARDs, such as Janus kinase inhibitors (JAKi), represent the most recent additions to the therapeutic arsenal available for RA management [6], 7].

According to the EULAR (European League Against Rheumatism) recommendations for the management of RA, the primary therapeutic goal is to achieve remission or, at the very least, a state of low disease activity. [6]. Despite the wide range of available therapies, between 20 and 40 % of patients do not respond to the first tsDMARD or bDMARD administered, and fewer than 50 % achieve clinical remission [8], 9]. Both national and European guidelines support early diagnosis and tighter control of inflammatory activity, following the treat-to-target strategy, which aims to achieve the therapeutic goal in the shortest possible time. As a result, an increasing number of patients are initiating treatment with biological agents or JAK inhibitors [10], 11].

The tsDMARDs currently approved for the treatment of RA include tofacitinib, baricitinib, upadacitinib, and filgotinib. These agents act as selective tyrosine kinase inhibitors targeting the Janus kinase (JAK) family. Their mechanism of action is based on the inhibition of cytokine- and growth factor-induced signal transduction, resulting in an immunosuppressive effect by blocking the activation of pro-inflammatory proteins involved in the pathophysiology of the disease. However, no biomarkers are currently available to identify which patients may benefit most from these treatments, making personalized therapeutic selection challenging. Although their safety profile is comparable to that of bDMARDs in most studies, some reports have raised concerns about the safety of these drugs in specific subgroups, prompting precautionary recommendations from regulatory agencies [12].

Several biomarkers have been proposed as potential predictors of remission in patients with RA [13], [14], [15]. However, to date, no robust biomarkers are available to predict response to the different types of treatments, which limits the possibility of personalized treatment selection [13], 16].

Although it is known that multiple immune cells are involved in the pathogenesis of RA, and that each cell type contributes differently to the inflammatory process, translating this knowledge into predictive clinical tools remains a challenge [17]. In this context, pharmacogenetics emerges as a promising and underexplored field, with the potential to identify genetic variants that could guide therapeutic selection and improve clinical outcomes. Therefore, the aim of this study is to analyze the association between pharmacogenetic variants and clinical response to JAK inhibitors in patients with RA.

## Materials and methods

### Study design

An observational, ambispective, single-center study was conducted at Hospital Universitario La Paz (LPUH) (Madrid, Spain). The study protocol was approved by the Institutional Ethics Committee (code PI-5318). Patients over 18 years of age with a confirmed diagnosis of RA, according to the classification criteria established by EULAR, were included. All participants had initiated or were in the process of initiating treatment with JAK inhibitors as part of their therapeutic regimen. Patients with concomitant immune-mediated diseases, such as systemic lupus erythematosus, spondyloarthritis, or other connective tissue disorders, were excluded to avoid interference in the assessment of therapeutic response. Sociodemographic and clinical data were collected from electronic medical records.

### Genotyping study

DNA extraction was performed from peripheral blood samples using the automated Maxwell^®^ system (Promega, Madison, USA), following the manufacturer’s instructions. Five genetic variants previously associated with response to biological therapies in RA patients were selected from the PharmGKB database (last accessed: September 29, 2025). These variants corresponded to the following genes: *FCGR2A* (rs1801274, C___9077561_20), *TNF* (rs1800629, C___7514879_10), *TLR2* (rs11938228, C__32212770_10), *TNFRSF10A* (rs20575, C__11852251_20), and *TNFRSF1B* (rs1061622, C___8861232_20).

Genotyping was performed using real-time PCR with TaqMan^®^ probes in OpenArray™ format, employing the QuantStudio™ 12K Flex Real-Time PCR System (Thermo Fisher Scientific). Amplification conditions included an initial denaturation phase at 93 °C for 10 min, followed by 50 cycles consisting of 45 s at 95 °C, 13 s at 94 °C, and 2 min at 53.5 °C, ending with a cooling step of 2 min at 25 °C [18].

### Data analysis

A descriptive analysis of the clinical characteristics of the study population was performed. Qualitative variables were summarized using absolute frequencies and percentages, while quantitative variables were expressed as mean and standard deviation. Normality of quantitative variables was assessed using the Kolmogorov–Smirnov test. The distribution of SNPs was evaluated for compliance with Hardy–Weinberg equilibrium (HWE) using a chi-squared test and minor allele frequencies were calculated directly from the observed genotypes in our cohort.

To analyze the association between the identified genetic biomarkers and response to JAK inhibitor treatment, the primary variable used was the Disease Activity Score-28 (DAS28). According to previously established criteria, based on DAS28 values, two dichotomous variables were defined: remission (DAS28≤2.6) vs. no remission (DAS28>2.6), and low disease activity (DAS28≤3.2) vs. moderate/high activity (DAS28>3.2) [19] PMID: 27762189. Patients with DAS28>3.2 were considered non-responders to the disease control objective. DAS28 was assessed at 6 and 12 months, as well as at the last available follow-up (final DAS28), using the last observation carried forward method.

The association between the studied genes and clinical variables of interest was examined using binary logistic regression models to estimate the relative risks of significant associations. All statistical tests were two-sided, with a significance level set at p<0.05. Analyses were performed using R software, version 4.3.3 (R Core Team, 2024).

## Results

### Characteristics of the JAKi-treated population

A total of 56 patients with RA who received JAK inhibitor therapy between January 2017 and March 2024 were included in the study. Baseline characteristics of the patients are presented in Table 1. The majority of patients were female (87.5 %) and were receiving concomitant treatment with methotrexate (66.07 %). The most commonly used JAK inhibitors in this population were tofacitinib and baricitinib.

**Table 1:** Characteristics of RA patients.

Characteristics	n=56
Sex	
Male	7 (12.5 %)
Female	49 (87.5 %)
Age at diagnosis, years (standard deviation)	42 (±11)
Smoking status	
Smoker	14 (25.0 %)
Former smoker	17 (30.4 %)
Non-smoker	25 (44.6)
ACPA	
Positive	50 (89.0 %)
Negative	5 (9.0 %)
Not available	1 (2.0 %)
FR	
Positive	46 (82.1 %)
Negative	10 (17.9 %)
JAK inhibitor	
Tofacitinib	20 (35.7 %)
Baricitinib	17 (30.4 %)
Upadacitinib	13 (23.2 %)
Filgotinib	6 (10.7 %)
Age at initiation of JAK inhibitor, years (standard deviation)	58 (±10)
Duration of treatment, months (standard deviation)	31 (±20)
Combination with methotrexate	
Yes	37 (66.1 %)
No	17 (30.4 %)
Not available	2 (3.5 %)
Treatment discontinuation	
Yes	27 (48.2 %)
No	29 (51.8 %)
DAS28 at 6 months	
Remission	20 (35.7 %)
Low activity	21 (37.5 %)
Moderate/high activity	11 (19.6 %)
Not available	4 (7.1 %)
DAS28 at 12 months	
Remission	16 (28.6 %)
Low activity	3 (5.3 %)
Moderate/high activity	14 (25.0 %)
Not available	23 (41.1 %)
DAS28 last observation	
Remission	15 (26.8 %)
Low activity	13 (23.2 %)
Moderate/high activity	22 (39.3 %)
Not available	6 (10.7 %)

Genotype distributions for each analyzed variant are presented in Table 2, together with allele frequencies in European non-Finnish populations according to gnomAD v4.1.0 and the minor allele frequencies (MAF) observed in our cohort. MAF values were calculated directly from the observed genotype data. The frequencies observed in our cohort are broadly consistent with those reported for reference European populations, although some SNPs show slight differences. None of the polymorphisms showed significant deviation from HWE (p>0.05), supporting the reliability of the genotyping results.

**Table 2:** Genotype counts, allele frequencies, and HWE assessment for analyzed SNPs.

Gene/variant	SNP ID	MAF (GenomaD)	MAF (LPUH)	WT	HET	HOM	HWE p-Value
*FCGR2A* NM_001136219.1:c.500A>G	rs1801274	0.5186	0.429	16	32	8	0.212
*TNF* NM_000594.3:c.-488G>A	rs1800629	0.1654	0.103	44	12	0	0.370
*TLR2* NM_001318789.1:c.-16-2098C>A	rs11938228	0.3634	0.208	34	16	3	0.549
*TNFRSF10A* NM_003844.3:c.626G>C	rs20575	0.4668	0.373	24	21	10	0.174
*TNFRSF1B* NM_001066.2:c.587T>G	rs1061622	0.2368	0.118	43	11	1	0.764

MAF, minor allelic frequencies; WT, wild types; HET, heterozygous variants; HOM, homozygous variants; HWE, Hardy-Weinberg equilibrium.

### Association between genetic polymorphisms and clinical effectiveness of JAK inhibitors

A statistically significant association was observed with the rs20575 variant of the *TNFRSF10A* gene in relation to the efficacy outcome of remission (Table 3). Specifically, patients carrying the CG or CC genotypes showed a higher likelihood of achieving remission at the end of the available follow-up period compared to those with the GG genotype (OR=4.47; 95 % CI: 1.182–22.128; p=0.039). This finding suggests that the presence of the C allele may increase the probability of achieving clinical remission by approximately 4.5 times. At 6 and 12 months, a higher percentage of patients with CG or CC genotypes were in clinical remission, although the differences did not reach statistical significance.

**Table 3:** Relationship between rs20575 genotype of the *TNFRSF10A* gene and remission.

	No remission (DAS28>2.6)	Remission (DAS28≤2.6)	OR (CI 95 %)	p-Value
*TNFRSF10A*-rs20575				

**6 months**				

GG CG/CC	14 (45 %) 17 (55 %)	8 (40 %) 12 (60 %)	1.235 (0.397–3.956)	0.716

**12 months**				

GG CG/CC	7 (39 %) 11 (61 %)	5 (31 %) 11 (69 %)	1.400 (0.340–6.058)	0.642

**Last observation**				

GG CG/CC	19 (53 %) 17 (47 %)	3 (20 %) 12 (80 %)	4.471 (1.182–22.128)	**0.039**

Bold values indicate statistically significant results (p<0.05).

When evaluating patients who had achieved at least low disease activity or clinical remission, a significant association was identified with the rs1801274 polymorphism of the *FCGR2A* gene at 12 months and at the last observation recorded assessment. Specifically, the presence of the AA genotype was significantly associated with a lower likelihood of achieving low disease activity or remission at 12 months [OR=0.074; 95 % CI=0.004–0.529; p=0.025] compared to AG or GG genotypes (Table 4). Clinically, this indicates that patients carrying the AA genotype have a 92.6 % reduced probability of achieving an adequate therapeutic response. Similarly, the AA genotype was significantly associated with a lower likelihood of achieving low disease activity at the last available follow-up [OR=0.2; 95 % CI=0.047–0.730; p=0.020] compared to AG or GG genotypes (Table 4). Therefore, the presence of the AA genotype appears to reduce the chances of a favorable therapeutic response, suggesting its potential role as a marker of reduced therapeutic efficacy in treatment with JAK inhibitors.

**Table 4:** Relationship between the rs1801274 genotype of the *FCGR2A* gene and low disease activity or remission.

	Moderate/high activity (DAS28>3.2)	Low activity/remission (DAS28≤3.2)	OR (CI 95 %)	p-Value
*FCGR2A*-rs1801274				

**6 months**				

GG/AG AA	7 (64 %) 4 (36 %)	30 (73 %) 11 (27 %)	0.642 (0.159–2.839)	0.537

**12 months**				

GG/AG AA	8 (57 %) 6 (43 %)	18 (95 %) 1 (5 %)	0.074 (0.004–0.529)	**0.025**

**Last observation**				

GG/AG AA	12 (54 %) 10 (46 %)	24 (86 %) 4 (14 %)	0.200 (0.047–0.730)	**0.020**

Bold values indicate statistically significant results (p<0.05).

No statistically significant associations were identified between clinical efficacy, assessed using remission or low disease activity, and the remaining genetic variants analyzed.

### Association of genetic polymorphisms with treatment discontinuation and survival of JAK inhibitors

**Figure 1:**
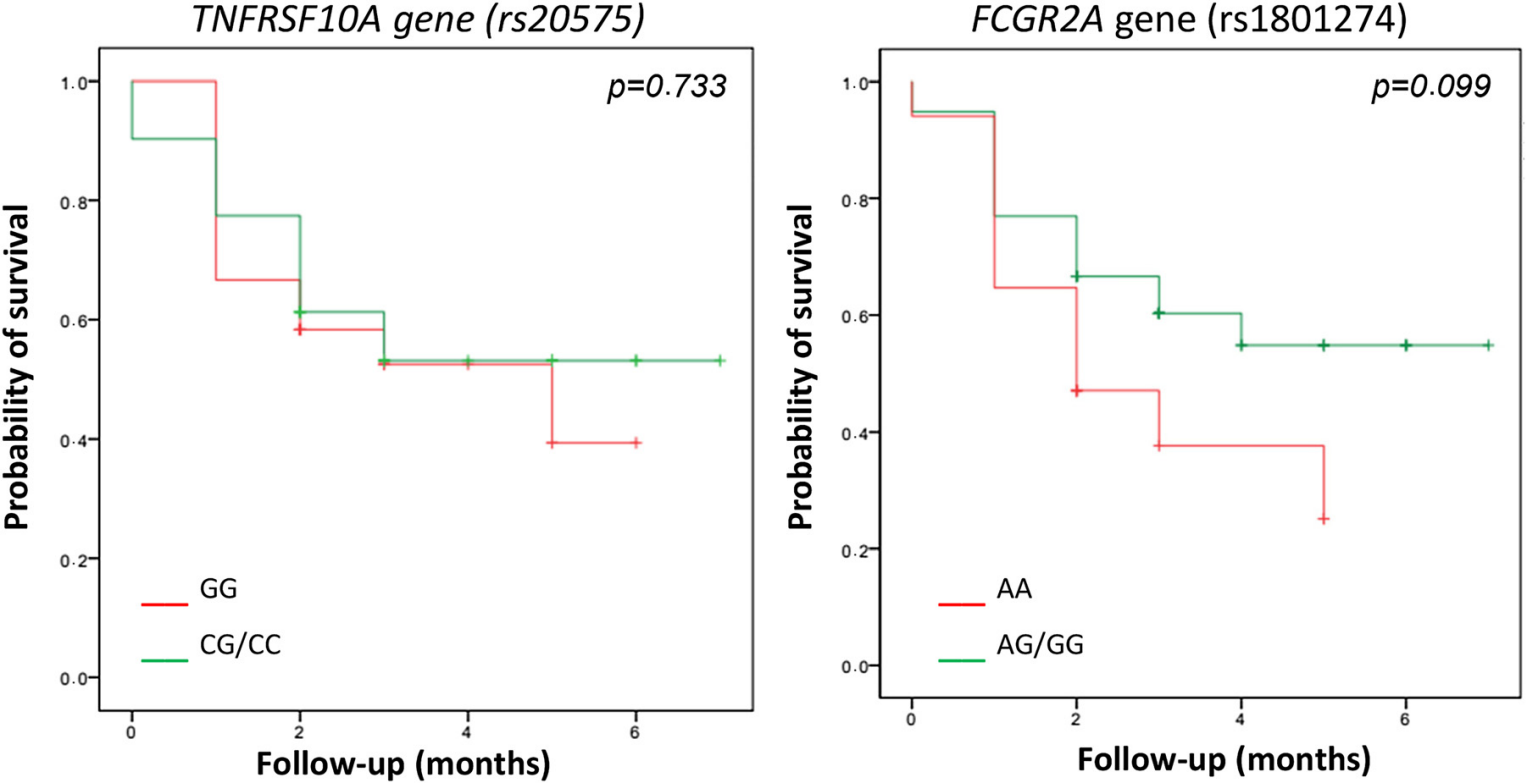
Kaplan–Meier survival analysis of time to treatment discontinuation stratified by rs20575 genotype in the *TNFRSF10A* gene and rs1801274 genotype in the *FCGR2A *gene.

In the overall cohort, 48 % of patients (27 out of 56) discontinued treatment during follow-up. The discontinuation rate was very similar among patients carrying the GG genotype of the *TNFRSF10A* gene (54.8 % continued vs. 45.2 % discontinued; p=0.721). In contrast, 65 % of patients with the AA genotype of the *FCGR2A* gene discontinued treatment (11 out of 17; p=0.103). In the survival analysis, no significant differences were observed in time to treatment discontinuation between carriers and non-carriers of the previously identified variants (Figure 1). However, patients with the AA genotype of the *FCGR2A* gene exhibited a tendency to discontinue JAK inhibitor treatment earlier, although this difference did not reach statistical significance (2.8±0.5 years vs. 4.6±0.5 years; p=0.09).

## Discussion

Our study is the first to evaluate the association between the genotype of genetic variants previously linked to response to biological therapies in a cohort of 56 patients treated with JAK inhibitors. Most patients included in the study were treated with either tofacitinib or baricitinib. The observed response to these treatments in our cohort was approximately 30 % for the remission target, and between 35 and 65 % of patients achieved at least low disease activity, depending on the time point evaluated.

In our study, individuals carrying the GG genotype of the rs20575 polymorphism in the *TNFRSF10A* gene showed a lower probability of achieving clinical remission in the medium to long term. The *TNFRSF10A* gene encodes the TRAIL-R1 receptor, a member of the TNF receptor family involved in both the induction of apoptosis and the production of inflammatory cytokines such as CCL-2 and IL-8 [20]. The first association of this polymorphism with RA was previously reported in a study involving 138 patients with RA and ankylosing spondylitis treated with a TNF inhibitor (infliximab), in which the presence of the G allele was linked to a lower treatment response after three months of follow-up [21]. Subsequent research confirmed that the best clinical response to biological agents such as adalimumab, etanercept, and infliximab in patients with rheumatoid arthritis and psoriatic arthritis was observed in individuals with the CC genotype [22]. Our findings suggest for the first time that the trend previously observed with TNF inhibitors may also apply in the context of JAK inhibitor treatment. However, additional studies with larger sample sizes are needed to confirm these observations. From a biological perspective, this association is consistent with a model in which JAK inhibitors attenuate cytokine-dependent cell survival and proliferation signals, and the CC or CG genotype at rs20575 may amplify this effect by promoting pro-apoptotic pathways and modulating the production of inflammatory cytokines. The potential interaction between genetic profile and the pharmacological action of JAK inhibitors could, therefore, facilitate the induction of clinical remission. Nevertheless, this interpretation should be considered a biologically plausible hypothesis, as no direct functional evidence is currently available to confirm it [23].

On the other hand, we identified that the presence of the AA genotype in the rs1801274 polymorphism of the *FCGR2A* gene was associated with a lower probability of achieving low disease activity, as well as with a non-significant trend toward earlier treatment discontinuation. To the best of our knowledge, this finding represents the first evidence of an association between this variant and JAK inhibitor treatment in patients with rheumatoid arthritis. The *FCGR2A* gene encodes the FcγRIIa receptor, which is expressed on the surface of various immune cells, including macrophages, neutrophils, and dendritic cells. This receptor plays a key role in antibody-mediated immune responses by facilitating the phagocytosis of immune complexes [24]. The rs1801274 genetic variant involves a substitution of histidine for arginine at position 131 in the second immunoglobulin-like domain of the FcγRIIa receptor. This modification affects its binding affinity to IgG2 antibodies. Specifically, the presence of histidine (A allele) enhances the inflammatory response and tissue damage, whereas the presence of arginine (absence of the A allele) reduces immune activation by decreasing immune complex clearance and is associated with a lower probability of developing RA [25]. In the context of our study, the increased immune system activation induced by the A allele may interfere with the effectiveness of JAK inhibitor treatment, which could explain the lower probability of achieving low disease activity in patients carrying the AA genotype.

In the case of biological therapies, the rs1801274 variant appears to have the opposite effect. It has previously been reported that in patients with rheumatoid arthritis treated with infliximab, the AA genotype was associated with greater improvement in the DAS28 compared to the GG genotype [26]. Similarly, individuals carrying the AA genotype showed a better treatment response at 12 months and a higher probability of achieving low disease activity at both 6 and 12 months in patients treated with abatacept [27], however, given the small cohort size, these findings should be interpreted with caution and require confirmation in larger studies The A allele has also been associated with greater treatment efficacy with adalimumab compared to the G allele [28]. In the case of rituximab, the AA genotype was also associated with a greater EULAR response at 6, 12, and 18 months, a higher remission rate at 6 months, and a significant improvement in DAS28 at 12 months [29]. These findings were confirmed in a subsequent meta-analysis. In that study, it was observed that caucasian patients carrying the A allele of the *FCGR2A* rs1801274 polymorphism may exhibit a better response to TNF inhibitor therapy. Specifically, it was suggested that the A allele of rs1801274 has lower binding affinity to IgG2, which would result in less efficient clearance of TNF inhibitors and, consequently, a more favorable therapeutic response [30].

Taken together, these data suggest that the rs1801274 polymorphism may serve as a useful pharmacogenetic marker for personalized therapeutic selection in RA. Specifically, patients with the AA genotype may benefit more from biological agents, while those with the GG genotype may show a better response to JAK inhibitor treatment. Nevertheless, further studies are required to confirm this hypothesis.

No statistically significant associations were identified for the remaining genetic variants analyzed. Although these variants have previously been linked to therapeutic response to biological treatments in diseases such as RA, psoriasis, and inflammatory bowel diseases [31], [32], [33], no studies have been found that associate them with treatment using JAK inhibitors in these conditions. This lack of association is consistent with the results obtained in our study, suggesting that the genetic profile involved in response to JAK inhibitors may differ from that associated with other biological therapies.

A key limitation of this study is the relatively small sample size, which precluded stratified analyses by JAK inhibitor subtype, particularly between more selective and less selective agents, and limited the ability to adjust for potential confounding variables. Another limitation is the lack of ethnicity data, which restricts direct comparisons with European reference populations. Despite this, a notable strength lies in the one year clinical follow-up, a critical period for capturing both adverse events and primary treatment failures.

This study provides new evidence supporting the relevance of pharmacogenetics in the personalization of rheumatoid arthritis therapy. For the first time, our findings suggest that specific genetic variants involved in immune regulation may play a significant role in modulating the therapeutic efficacy of JAK inhibitors. While these results offer promising perspectives for refining treatment strategies within the framework of precision medicine, they should be interpreted with caution and require validation in larger, well-characterized cohorts.
